# Construction of three‐gene‐based prognostic signature and analysis of immune cells infiltration in children and young adults with B‐acute lymphoblastic leukemia

**DOI:** 10.1002/mgg3.1964

**Published:** 2022-05-23

**Authors:** Chunli Xiang, Jie Wu, Liang Yu

**Affiliations:** ^1^ Department of Hematology Huai'an First People's Hospital Affiliated to Nanjing Medical University Huai'an China; ^2^ Key Laboratory of Hematology of Nanjing Medical University Nanjing China; ^3^ Department of Emergency Medicine The Fifth People's Hospital of Huai'an Huai'an China

**Keywords:** B‐acute lymphoblastic leukemia, bioinformatical analysis, differentially expressed genes, immune cells infiltration, prognostic signature

## Abstract

**Background:**

Although B‐acute lymphoblastic leukemia (B‐ALL) patients' survival has been improved dramatically, some cases still relapse. This study aimed to explore the prognosis‐related novel differentially expressed genes (DEGs) for predicting the overall survival (OS) of children and young adults (CAYAs) with B‐ALL and analyze the immune‐related factors contributing to poor prognosis.

**Methods:**

GSE48558 and GSE79533 from Gene Expression Omnibus (GEO) and clinical sample information and mRNA‐seq from Therapeutically Applicable Research to Generate Effective Treatments (TARGET) database were retrieved. Prognosis‐related key genes were enrolled to build a Cox proportional model using multivariate Cox regression. Five‐year OS of patients, clinical characteristic relevance and clinical independence were assessed based on the model. The mRNA levels of prognosis‐related genes were validated in our samples and the difference of immune cells composition between high‐risk and low‐risk patients were compared.

**Results:**

One hundred and twelve DEGs between normal B cells and B‐ALL cells were identified based on GSE datasets. They were mainly participated in protein binding and HIF‐1 signaling pathway. One hundred and eighty‐nine clinical samples were enrolled in the study, both Kaplan–Meier (KM) analysis and univariate Cox regression analysis showed that *CYBB*, *BCL2A1*, *IFI30*, and *EFNB1* were associated with prognosis, *CYBB*, *BCL2A1*, and *EFNB1* were used to construct prognostic risk model. Moreover, compared to clinical indicators, the three‐gene signature was an independent prognostic factor for CAYAs with B‐ALL. Finally, the mRNA levels of *CYBB*, *BCL2A1*, and *EFNB1* were significantly lower in B‐ALL group as compared to controls. The high‐risk group had a significantly higher percentage of infiltrated immune cells.

**Conclusion:**

We constructed a novel three‐gene signature with independent prognostic factor for predicting 5‐year OS of CAYAs with B‐ALL. Additionally, we discovered the difference of immune cells composition between high‐risk and low‐risk groups. This study may help to customize individual treatment and improve prognosis of CAYAs with B‐ALL.

## Graphical abstract

We constructed a novel three‐gene signature with independent prognostic factor for predicting 5‐year OS in CAYAs with B‐acute lymphoblastic leukemia. Additionally, we discovered the difference of immune cells composition between high and low‐risk group. This study may help to customize individual treatment and improve prognosis of CAYAs B‐ALL patients.

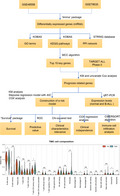

## 1 INTRODUCTION

B‐acute lymphoblastic leukemia (B‐ALL) is the most prevalent hematological malignancy in children (Filbin & Monje, 2019; Inaba & Mullighan, 2020). Current treatments including chemotherapy, radiotherapy, targeted therapy, immune therapy, Chimeric Antigen Receptor T‐Cell Immunotherapy (CAR‐T), and allogenic hematopoietic stem cell transplantation (allo‐HSCT) are effective with the cure rates approaching 90% for children, and the outcome has improved for young adults with the application of pediatric‐inspired regimens, but some patients especially young adults who experienced relapse remains dismal (Jabbour et al., 2018; Malard & Mohty, 2020; Pui, 2020). Relapsed ALL remains the major cause of cancer‐related deaths, the 5‐year overall survival (OS) rates for relapsed ALL remain between 25% and 40% (Oskarsson et al., 2016). For many B‐ALL patients, relapse was caused by the chemotherapy resistance (Huang et al., 2020), while chemotherapy resistance mainly due to molecular abnormalities, such as genetic, epigenetic, transcriptomic, and proteomic alterations, these molecular abnormalities could be useful for better risk stratification and predicting treatment response of patients (Aberuyi et al., 2019).

Currently, prognostic factors of B‐ALL are mainly focused on clinical factors, genetic alterations, and minimal residual disease (MRD) for risk stratification (Inaba & Mullighan, 2020). Clinical factors containing age (infant or ≥ 10 years), white blood cell (WBC) count at diagnosis (≥50 × 10^9^/L), central nervous system (CNS) or testicular involvement, race (Hispanic or black), and male sex have been considered adverse prognostic factors (El Ashry et al., 2021; Inaba & Mullighan, 2020; Lee et al., 2021). Genetic alterations such as low hypodiploid, positive *E2A‐PBX1*, or *KMT2A* rearrangement, Philadelphia chromosome‐positive and Ph‐like, etc. have been also recognized as worse prognostic factors (Inaba & Mullighan, 2020; Lee et al., 2021). In addition, positive MRD at end of remission induction was associated with an unfavorable prognosis. Recently, large researches indicated that telomerase activity, dysregulated microRNAs, and even long non‐coding RNA were regarded as prognostic factors of B‐ALL (Gao, 2021; Karow et al., 2021; Rashed et al., 2019). However, few studies were reported that using some genes mRNA expression levels as 5‐year OS prognostic biomarkers especially for CAYAs with B‐ALL.

Gene Expression Omnibus (GEO) is an open‐access data that stores microarray, next‐generation sequencing, high‐throughput sequencing data, and clinical information. Using this database, we can retrieve some experimental sequencing data (Barrett et al., 2013; Das et al., 2020). Additionally, Therapeutically Applicable Research To Generate Effective Treatments (TARGET) is a database specifically for childhood tumors, it contains ALL, Acute Myeloid Leukemia (AML), Kidney Tumors, Neuroblastoma and Osteosarcoma, and administrated by NCI's Office of Cancer Genomics and Cancer Therapy Evaluation Program, it has the right powerful and more targeted (Lyu et al., 2019). TARGET program contains raw genomic data as well as diagnostic, histologic, and clinical outcome data (Grossman et al., 2016). Therefore, to explore new genes that related to the prognosis of CAYAs with B‐ALL and increase credibility of the result, GEO and TARGET databases were analyzed integratively.

Here, the main aim of this study was to identify the prognosis‐related novel genes and construct a credible gene‐based prognostic model of CAYAs with B‐ALL using the two databases. Then, immune‐related factors contributing to poor prognosis were also analyzed. This may help develop a personalized treatment to improve the survival of these patients.

## 2 MATERIALS AND METHODS

### 2.1 Data collection

Two gene expression profiles (GSE48558 and GSE79533) from the GEO database (http://www.ncbi.nlm.nih.gov/geo/) were extracted (Barrett et al., 2013), the clinical sample information and mRNA‐seq (ALL Phase II) were obtained from the TARGET database (https://ocg.cancer.gov/programs/target). The platforms of GSE48558 (Cramer‐Morales et al., 2013) and GSE79533 (Hirabayashi et al., 2017) were GPL6244 and GPL570, respectively. The gene expression data of 38 samples (11 normal B cells and 27 children B‐ALL cells) from GSE48558 and 229 samples (three normal B cells and 226 children B‐ALL cells) from GSE79533 were screened. The clinical samples lacking survival status or OS time and alive patients with follow‐up less than 5 years were excluded. The samples with mRNA expression levels, survival status and OS time were retained. Ultimately, a total of 189 CAYAs with B‐ALL samples were enrolled in this study. The specific screening process is shown in the Figure S1.

### 2.2 DEGs identification

DEGs between normal B cells and children B‐ALL cell samples from two GSE datasets were analyzed by R software “limma” package (http://www.bioconductor.org/packages/release/bioc/html/limma.html). To select significant DEGs, the data were standardized and filtered. *p*‐value < .05 and |log2 Fold Change (FC)| ≥ 1 were set as the threshold. Furtherly, based on the threshold: log2FC ≥1, DEGs were defined as upregulated genes; correspondingly, log2FC ≤ −1, DEGs were defined as downregulated genes. Subsequently, upregulated or downregulated overlapped genes from two datasets were defined as co‐upDEGs or co‐downDEGs, co‐DEGs were displayed with the online Venn diagram tool (available online: https://bioinfogp.cnb.csic.es/tools/venny/index.html).

### 2.3 GO and KEGG pathway enrichment analysis

Gene ontology (GO) and Kyoto Encyclopedia of Genes and Genomes (KEGG) pathway enrichment analysis were conducted using the online databases KOBAS 3.0 (http://kobas.cbi.pku.edu.cn). GO terms and KEGG pathways were analyzed based on *p* value (corrected *p* < .05 and *p* < .05, respectively).

### 2.4 Protein–protein interaction (PPI) construction and key gene extraction

Co‐DEGs were uploaded to the STRING online website version 11.0 (http://www. string‐db.org/), which is a PPI network functional enrichment analysis tool. To acquire the network graph of PPI, an interaction score > 0.4 was set as the threshold used for analysis. The Maximal Clique Centrality (MCC) algorithm, one of the most useful methods to select essential proteins from PPI networks (Chin et al., 2014), was used to identify key genes in the cytoHubba plugin of Cytoscape.

### 2.5 Prognosis and survival analysis

B‐ALL clinical samples information and mRNA‐seq from TARGET data is open to the public. To obtain information related to B‐ALL survival, 189 clinical samples with information including survival status, OS time and mRNA expression levels were enrolled. R software “Survival” package (https://CRAN.R‐project.org/package=survival), univariate Cox survival analysis and Kaplan–Meier (KM) survival analysis methods were applied to investigate the association between genes expression levels and the OS of patients. A value of *p* < .05 was the cutoff value to identify significant prognosis‐related genes.

### 2.6 Prognostic risk score model construction and clinical characteristics relevance evaluation

Genes related to survival were obtained through the univariate Cox regression analysis and KM analysis, then three key genes *CYBB*(OMIM#300481), *BCL2A1*(OMIM#601056), and *EFNB1*(OMIM#300035) were used to establish model via stepwise regression Akaike Information Criterion (AIC) method, finally prognosis‐related gene signature was constructed via multivariate Cox regression analysis. Based on the Cox regression risk model coefficients, the risk score for each patient was calculated.

$$\begin{array}{c} \text{Risk score}=\text{coef}\left(\text{gene1}\right)\times \text{expr}\left(\text{gene1}\right)+\text{coef}\left(\text{gene2}\right)\times \text{expr}\left(\text{gene2}\right)\\ \;+\text{coef}\left(\text{gene3}\right)\times \text{expr}\left(\text{gene3}\right) \end{array}$$

Coef (gene) was defined as the coefficient of gene correlated with survival. Expr (gene) was defined as the gene mRNA expression levels.

The 189 patients were divided into low‐risk and high‐risk groups based on the median risk score. Finally, the predictive efficiency of the risk signature was evaluated using time‐dependent receiver operating characteristic (ROC) curve. Chi‐square test was used to analyze the relation between risk score and clinical factors. Cox proportional hazards regression analysis was used to estimate the independence of the prognostic value of signature.

### 2.7 Specimen collection and cell culture

Three peripheral blood (PB) samples got from healthy children; B‐ALL cells Nalm‐6, purchased from the Chinese Academy of Sciences (Shanghai, China). RS4;11 and SUP‐B15, obtained from American Type Culture Collection (USA). Nalm‐6 and RS4;11 cell lines were cultured in RPMI‐1640 medium supplemented with 10% fetal bovine serum (FBS), 1% penicillin/streptomycin at 37°C in a humidified atmosphere containing 5% CO_2_. SUP‐B15 cell line was cultured in Iscove's modified Dulbecco's medium (IMDM) medium supplemented with 20% FBS, 1% penicillin/streptomycin in humidified atmosphere of 5% CO_2_ at 37°C.

### 2.8 Quantitative reverse transcription polymerase chain reaction (qRT‐PCR)

Total RNAs of PB mononuclear cells from three healthy children and B‐ALL cells were extracted using TRIzol reagent (VICMED, China) following the manufacturer's protocol. Then, the purity and concentration of the isolated RNA were measured using NanoDrop 2000c (Thermo Fisher Scientific, USA). cDNA was synthesized through Reverse Transcriptase Kits (Vazyme, China) containing gDNA eraser (DNAse) following the manufacturer's instructions. The qRT‐PCR reactions of *GAPDH* (OMIM#138400, NM_001256799.3), *CYBB* (NM_000397.4), *BCL2A1* (NM_001114735.2), and *EFNB1* (NM_004429.5) were performed using 7500 Fast Real‐Time PCR System (Applied Biosystems, CA). Relative mRNA levels were calculated via the 2^‐ΔΔCT^ method. Primer sequences obtained from Sangon Biotech (Shanghai, China) are listed in Table 1.

**TABLE 1:** Primer sequences

Gene	Primer sequences (F: forward; R: reverse)
*CYBB*	F: 5′‐AAGATGCGTGGAAACTACCTAA‐3′
	R: 5′‐TTTTTGAGCTTCAGATTGGTGG‐3′
*BCL2A1*	F: 5′‐AGAATCTGAAGTCATGCTTGGA‐3′
	R: 5′‐CTCCTTTTCCATCACTTGGTTG‐3′
*EFNB1*	F: 5′‐CAGAGCAGGAAATACGCTTTAC‐3′
	R: 5′‐AATCATGGTGCTTCTTGAACTC‐3′
*GAPDH*	F: 5′‐CTGGGCTACACTGAGCACC‐3′
	R: 5′‐AAGTGGTCGTTGAGGGCAATG‐3′

Abbreviations: *BCL2A1*, NM_001114735.2; *CYBB*, NM_000397.4; *EFNB1*, NM_004429.5; *GAPDH*, NM_001256799.3.

### 2.9 Abundance analysis of immune cells

CIBERSORT (https://cibersort.stanford.edu/) was used to evaluate the composition of 22 immune cell types between low‐risk and high‐risk groups.

### 2.10 Statistics

All statistics were performed in R software (version 4.0.5) and IBM SPSS Statistics (version 21.0). Measurement data of three experiments were expressed as mean ± standard deviation. Student's *t* test was used to evaluate difference of data between two groups, and the chi‐square test was applied to assess categorical variables. The Cox proportional hazards regression model was used to analyze the prognostic value of each parameter. A value of *p* < .05 was considered statistically significant.

## 3 RESULTS

### 3.1 DEGs between normal B cells and children B‐ALL cells

**FIGURE 1:**
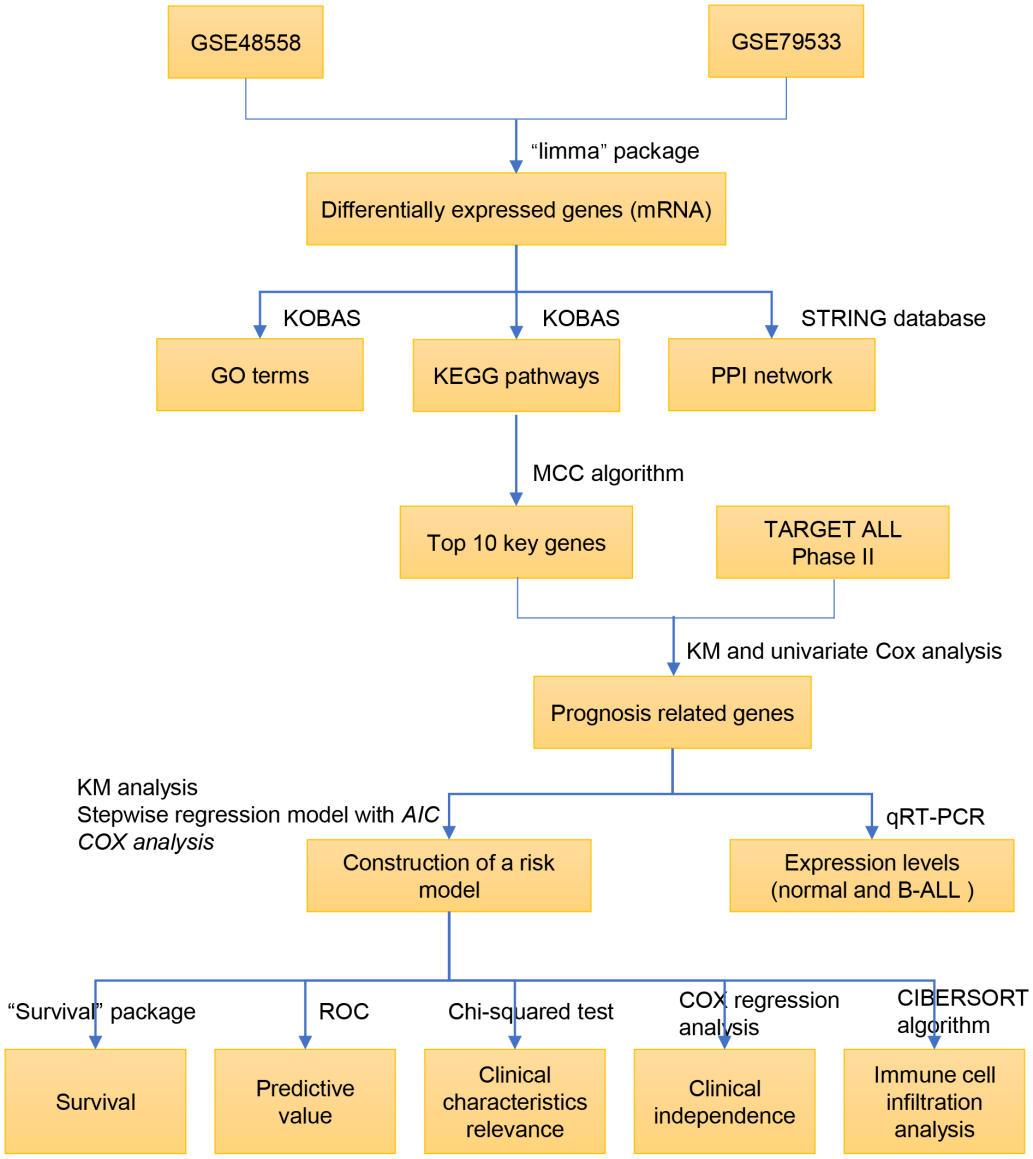
The flow chart of data processing in this work

**FIGURE 2:**
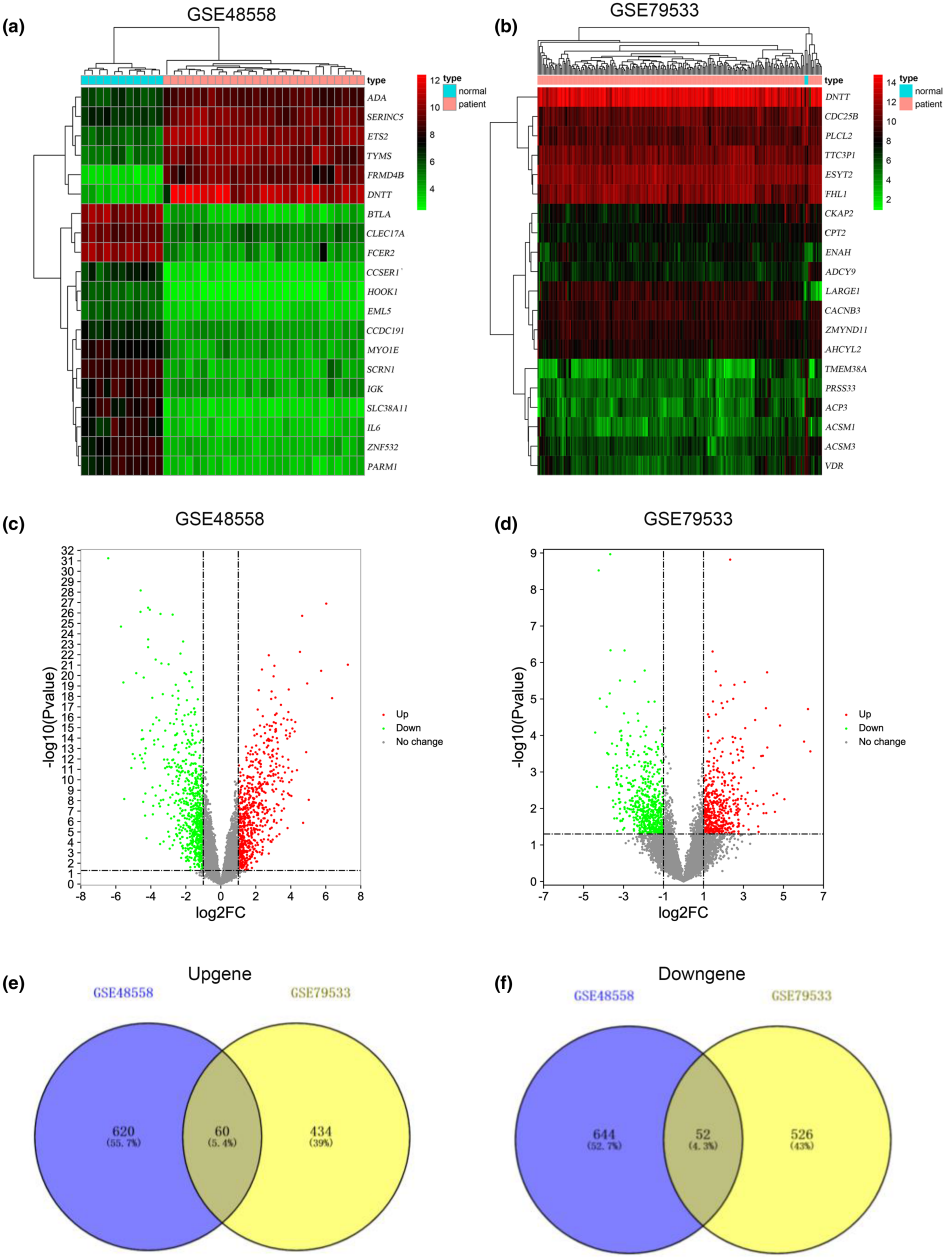
DEGs Identification. (a–b) Heatmap exhibited top 20 significant DEGs from GSE48558 and GSE79533 datasets. (c–d) Volcano map showed all significant DEGs from GSE48558 to GSE79533 datasets. (e–f) Authentication of 112 co‐DEGs in GSE48558 and GSE79533 datasets. Purple and yellow circles meant GSE48558 and GSE79533 datasets, respectively

To illustrate the study clearly, a flow diagram was designed to elucidate the research (Figure 1). The two datasets GSE48558 and GSE79533 were collected from the GEO database. All gene expression values were standardized, gene expression values of GSE79533 were log2 converted. Then, gene expression values of normal B cells and children B‐ALL cells were analyzed by R software “limma” package. Six hundred and eighty‐two and 494 significantly upregulated DEGs were screened from the GSE48558 and GSE79533 datasets respectively, correspondingly 696 and 578 significantly downregulated DEGs. Cutoff standard was *p*‐value .05, |log 2FC|) ≥ 1. The top significant 20 DEGs from the GSE48558 and GSE79533 were displayed in heatmap (Figure 2a,b), and all DEGs were shown in Volcano plots (Figure 2c,d). One hundred and twelve co‐DEGs containing 60 upregulated and 52 downregulated genes were screened by visual analysis method of Venn diagram (Figure 2e,f).

### 3.2 GO analysis and KEGG pathway enrichment analysis of DEGs

**FIGURE 3:**
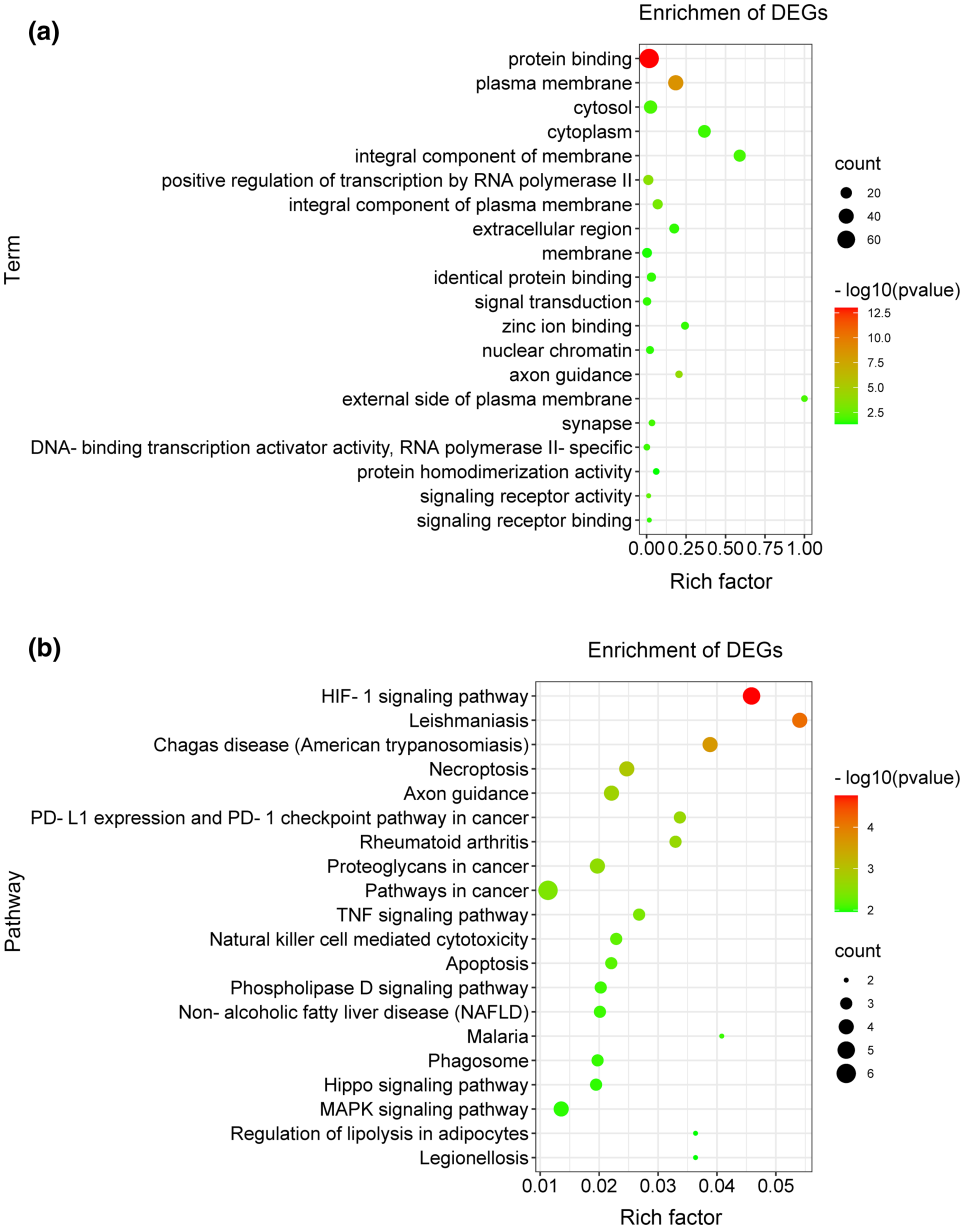
Enrichment analysis of top 20 significant co‐DEGs. (a) GO analysis illustrated the functional pathway. (b) Top 20 pathway enrichment was displayed by KEGG analysis

GO and KEGG analysis of DEGs were performed using KOBAS 3.0. The top 20 enrichments analysis are shown in Figure 3a,b. GO terms of DEGs were mostly located in plasma membrane, cytosol, cytoplasm, integral component of membrane, and involved in protein binding and positive regulation of transcription by RNA polymerase II (Figure 3a); while KEGG pathways of DEGs were mostly as follows: HIF‐1, PD‐1 checkpoint, TNF, Phospholipase D, Hippo, and MAPK (Figure 3b).

### 3.3 PPI network and key genes

**FIGURE 4:**
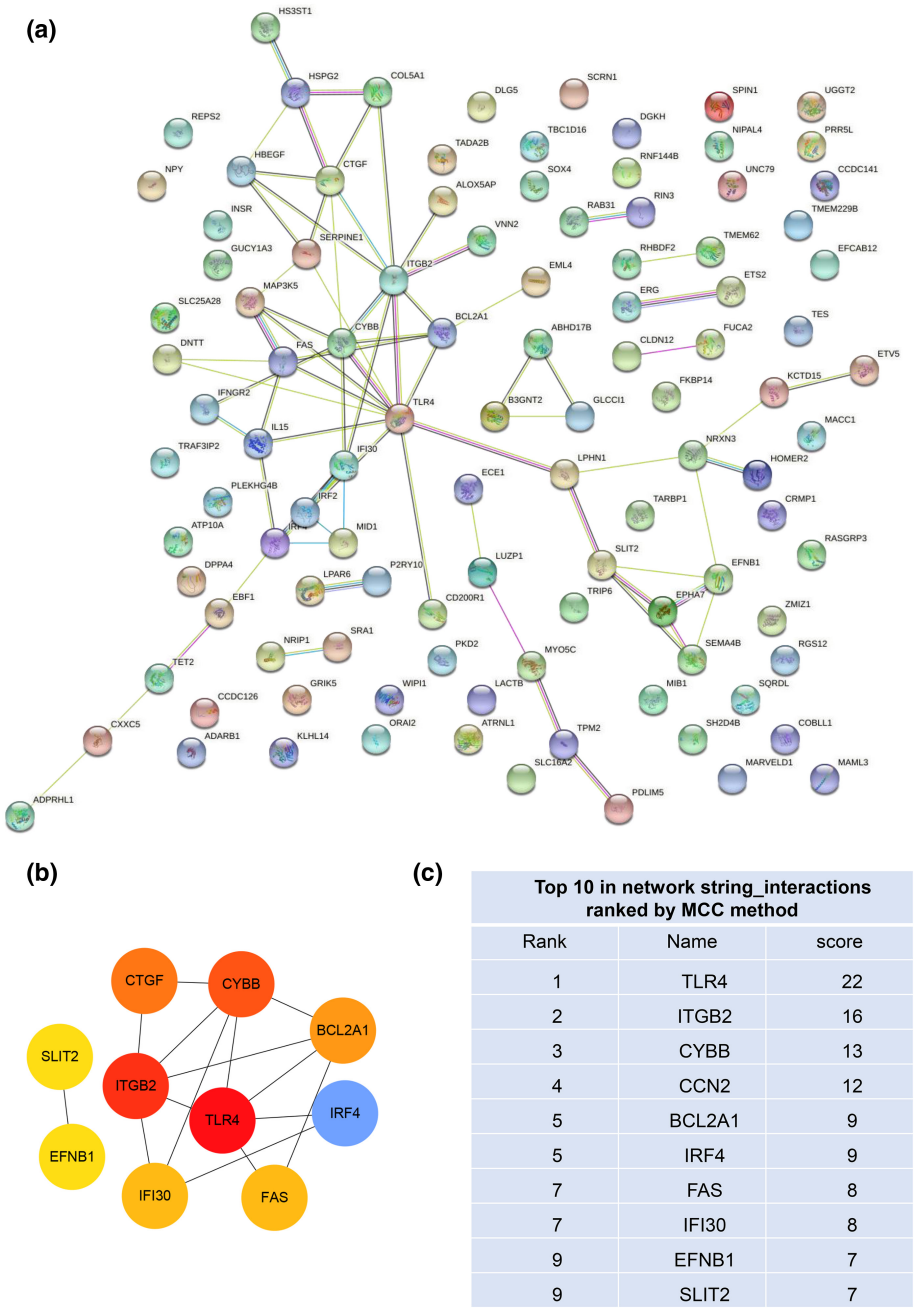
PPI network and key genes. (a) All co‐DEGs were constructed by STRING online database, the confidence level was greater than or equal to 0.4. (b–c) Top 10 key genes and their scores were calculated by MCC method

In order to select key genes, 112 co‐DEGs were analyzed using the PPI network in STRING online database and Cytoscape software (Figure 4a). Subsequently, they were calculated using MCC calculation method in the cytoHubba plugin of Cytoscape, 10 key genes (*TLR4*, *ITGB2*, *CYBB*, *CCN2*, *BCL2A1*, *IRF4*, *FAS*, *IFI30*, *EFNB1*, and *SLIT2*) with highest MCC scores were obtained (Figure 4b,c).

### 3.4 Key genes related to survival

**FIGURE 5:**
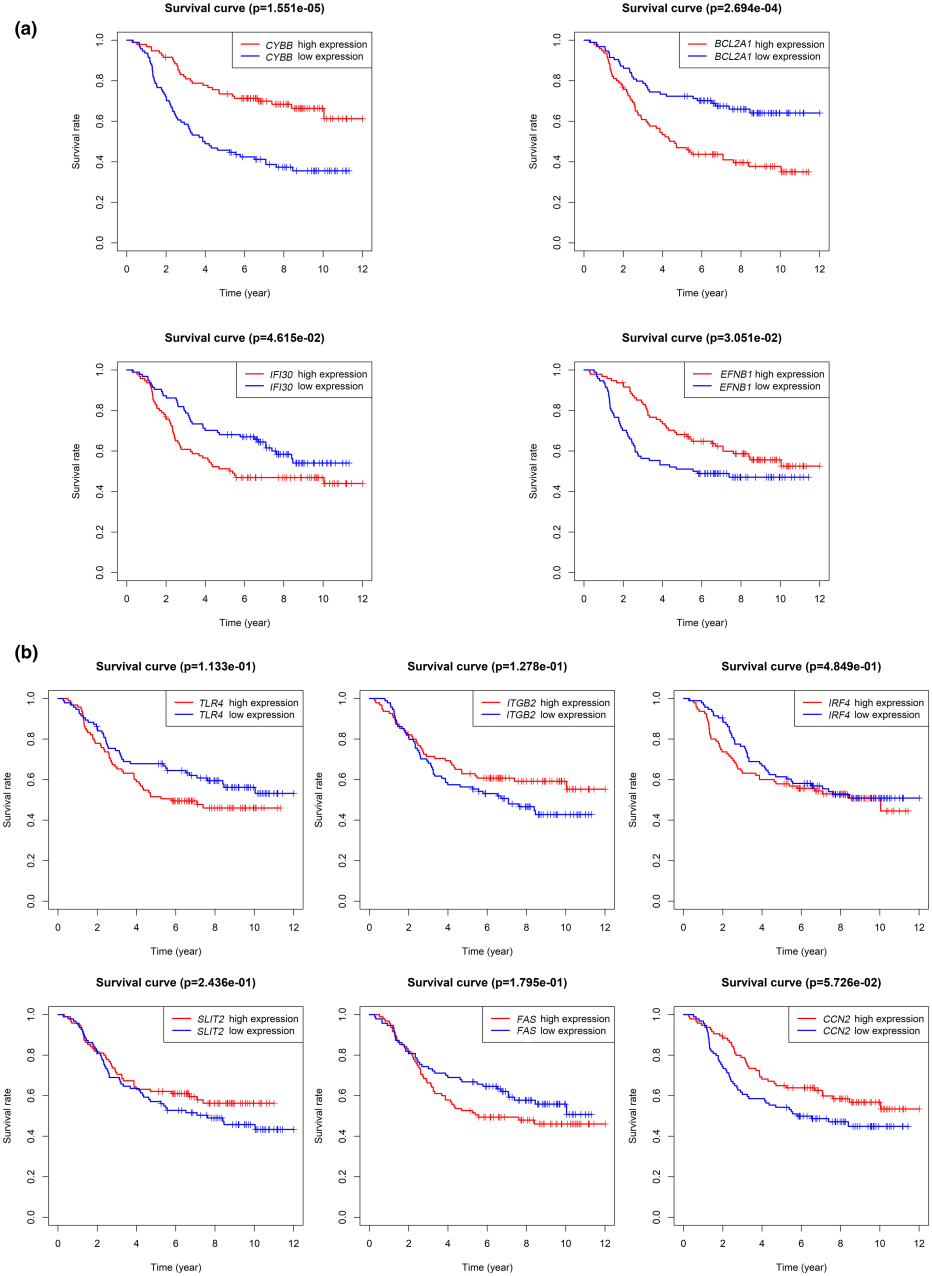
KM survival analysis of 10 key genes. (a) CYBB, BCL2A1, IFI30, and EFNB1 were associated with survival (*p* < .05); (b) TLR4, ITGB2, IRF4, SLIT2, FAS and CCN2 were not related to survival (*p* > .05)

Based on the clinical information and mRNA‐seq expression data from TARGET data matrix, 10 key genes associated with the 5‐year OS of patients were explored using KM survival analysis and univariate Cox survival analysis, respectively. KM survival analysis revealed that four genes (*CYBB*, *BCL2A1*, *IFI30*, and *EFNB1*) were associated with 5‐year OS (*p* < .05, Figure 5a), but other six genes were not (*p* > .05, Figure 5b); Univariate Cox survival analysis identified seven key genes (*CYBB*, *CCN2*, *BCL2A1*, *FAS*, *IFI30*, *EFNB1*, and *SLIT2*) were associated with 5‐year OS (*p* < .05, Table 2). Therefore, overlapped genes *CYBB*, *BCL2A1*, *IFI30*, and *EFNB1* identified by the two survival analysis methods were regarded as the key genes related to prognosis.

**TABLE 2:** Univariate Cox survival analysis

Gene	HR	*p* value
*TLR4*	1.063833	.173326
*ITGB2*	0.959475	.561492
*CYBB*	0.84713	.000332
*CCN2*	0.907505	.01326
*BCL2A1*	1.229886	.000125
*IRF4*	1.121806	.087944
*FAS*	1.224464	.009774
*IFI30*	1.225176	.000764
*EFNB1*	0.780803	2.21E‐05
*SLIT2*	0.939958	.036937

Abbreviation: HR, hazard ratio.

### 3.5 Multivariate Cox regression analysis and construction of risk score signature

Based on survival status, OS time and the mRNA expression levels of genes from 189 patients, three key prognosis‐related genes were selected to construct the prognostic‐related gene signature with stepwise method using the AIC. Finally, a predictive model for 5‐year OS in CAYAs with B‐ALL was prosed, risk score = expr (*BCL2A1*) × 0.193929‐expr(*EFNB1*) × 0.22053‐expr(*CYBB*) × 0.14097 (Table 3). In 189 clinical samples, cases with risk scores less than or equal to 0.877765(median risk score) were defined as low‐risk group, others were defined as high‐risk group.

**TABLE 3:** Multivariate Cox regression analysis for *CYBB*, *BCL2A1,* and *EFNB1*

Gene	Coefficient	HR	SE(coefficient)	*Z*	*p* value
*EFNB1*	−0.22053	0.80209	0.055081	−4.00383	6.23E‐05
*BCL2A1*	0.193929	1.21401	0.053377	3.633161	.00028
*CYBB*	−0.14097	0.868517	0.044627	−3.1588	.001584

Abbreviation: HR, hazard ratio.

### 3.6 Predictive capability and efficiency of the prognostic signature

**FIGURE 6:**
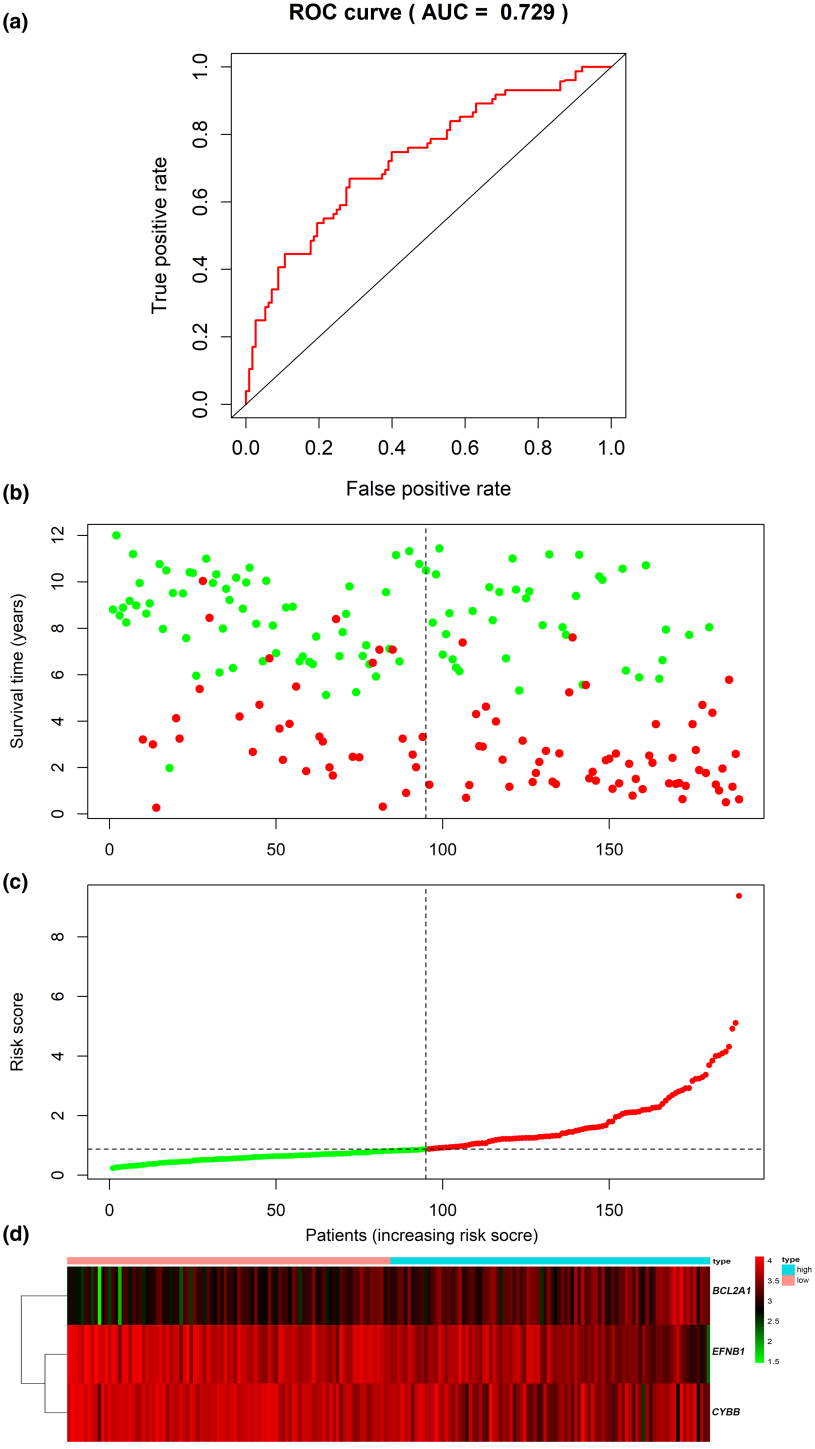
Assessment of the prognosis risk model. (a) The 5‐year ROC curves in the 189 clinical samples from the TARGET dataset. (b) Survival status and survival time of patients in low‐risk and high‐risk groups. Red and green meant living and dead patients, respectively. (c) Patients' risk scores in the low‐risk and high‐risk groups. Red and green meant living and dead patients, respectively. (d) BCL2A1, EFNB1, and CYBB mRNA expression levels in each patient

To evaluate the predictive value and efficiency of the prognostic signature, ROC curve was drawn (Figure 6a). Area under the curve (AUC) was used to assess the discriminative ability of prediction models, in the 189 samples, AUC of the risk signature 5‐year OS was 0.729. In addition, the patients were classified into low‐risk and high‐risk groups based on the median risk score. The result illustrated that high‐risk patients had lower OS compared to low‐risk patients. (Figure 6b–d; Table 4).

**TABLE 4:** Assessment of 5‐year OS in two groups

Group	OS (%)	Lower 95%	Upper 95%
High risk	44.7	35.7	56
Low risk	74.5	66.2	83.9

Abbreviation: OS, overall survival rate.

### 3.7 Analysis of the relation between risk score and clinical characteristics and Evaluation of the independence of the three‐gene‐based prognostic signature

**FIGURE 7:**
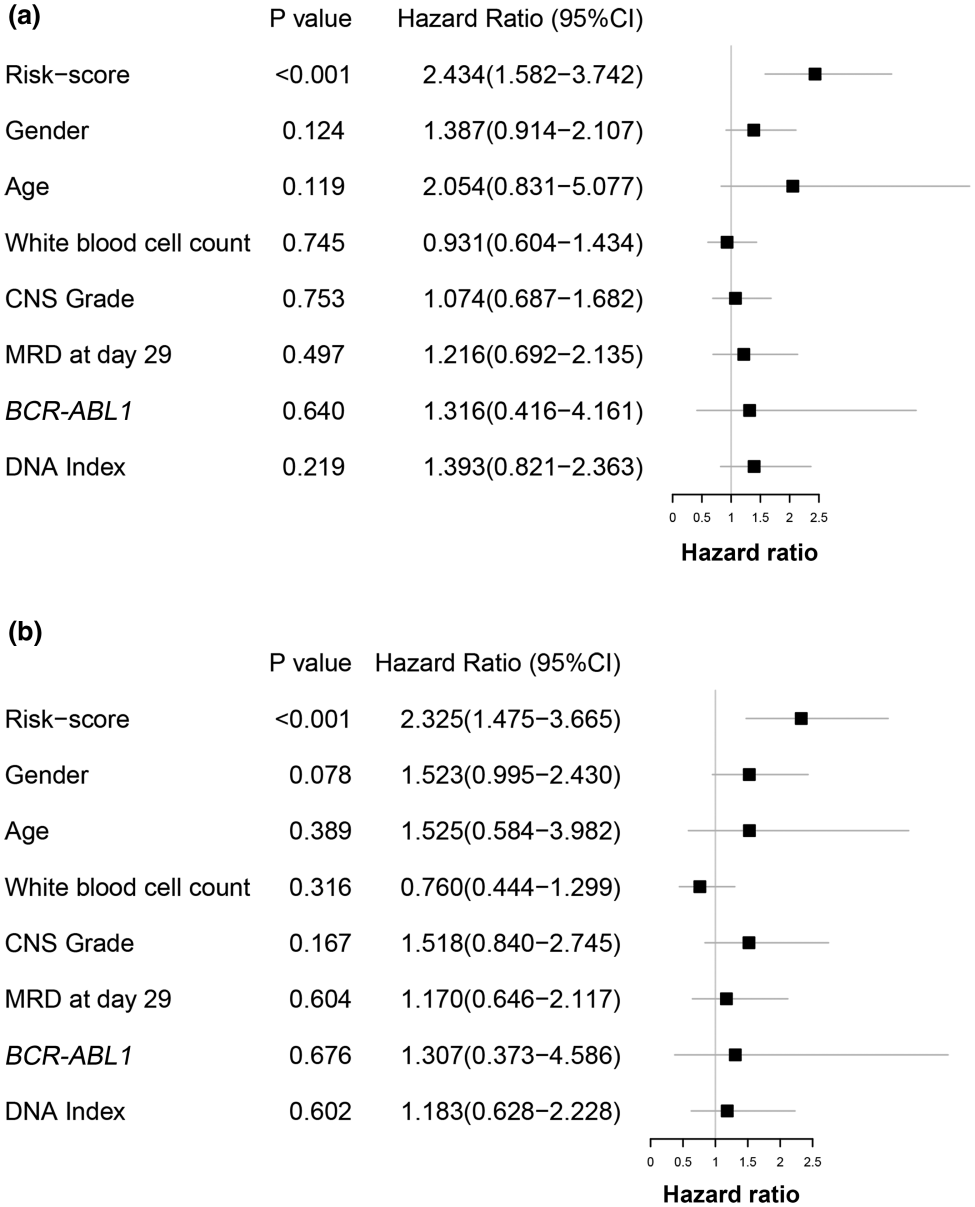
The independency of the risk model was assessed based on univariate Cox regression analysis (a) and multivariate Cox regression analysis (b) Bar length represented variable of Hazard ratio 95% CI

To investigate whether any correlation existed between risk score and clinical characteristics, we compared risk score with different clinical features. The result demonstrated that no significant differences were found between high‐risk and low‐risk groups in terms of age, gender, WBC count, CNS grade, MRD at day 29, *BCR‐ABL1* and DNA Index (*p* < .05; Table 5). To furtherly evaluate the independence of the signature for predicting 5‐year OS, we performed univariate and multivariate Cox regression analysis to calculate *p* value and hazard ratio in terms of risk score and different clinical characteristics. The result of two analysis methods both indicated that age, gender, WBC count, CNS grade, MRD, *BCR‐ABL1*, and DNA Index were all not significantly associated with the 5‐year OS of CAYAs with B‐ALL, while risk score was significantly related to the 5‐year OS and was an independent prognostic factor (Figure 7a,b; univariate *p* < .001, HR 2.434; multivariate *p* < .001, HR 2.325).

**TABLE 5:** Correlation between risk score and clinical characteristics

Characteristic	Group		C^2^	*p* value
	High risk	Low risk		
**Gender**			0.641	.423
Female	44(46.81%)	50(53.19%)		
Male	50(52.63%)	45(47.37%)		
**Age**			1.411	.235
<18	89(48.90%)	93(51.10%)		
≥18	5(71.43%)	2(28.57%)		
**WBC count**			0.043	.837
<50 × 10^9^ ↑/L	59(49.17%)	61(50.83%)		
>50 × 10^9^ ↑/L	35(50.72%)	34(49.28%)		
**CNS grade**			0.370	.831
CNS 1	75(49.67%)	76(50.33%)		
CNS 2	17(48.57%)	18(51.43%)		
CNS 3	2(66.67%)	1(33.33%)		
**MRD at Day 29**			1.274	.529
<0.01%	19(52.78%)	17(47.22%)		
≥0.01%	63(47.37%)	70(52.63%)		
unknown	12(60.00%)	8(40.00%)		
***BCR‐ABL1***			0.000	1.0
Negative	92(50%)	92(50%)		
Positive	2(40.00%)	3(60.00%)		
**DNA index**			0.144	.705
1–1.16	81(50.31%)	80(49.69%)		
>1.16	13(46.43%)	15(53.57%)		

Abbreviations: C^2^, chi‐square; CNS, central nervous system; MRD, minimal residual disease; WBC, white blood cell.

### 3.8 RNA expression levels detection

**FIGURE 8:**
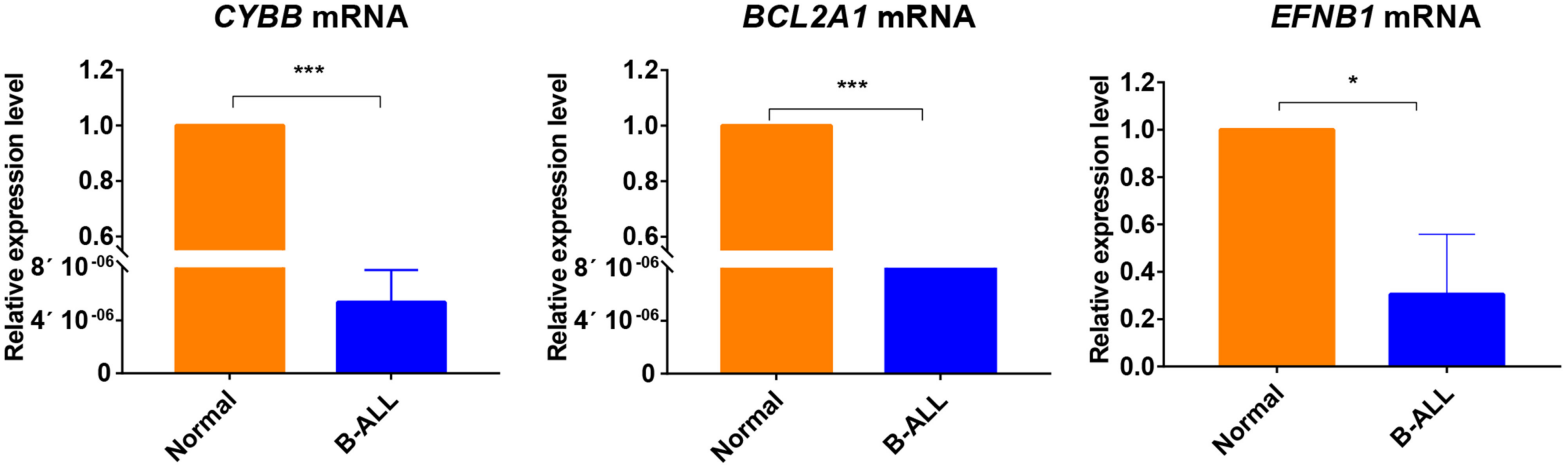
Relative RNA expression levels of CYBB, BCL2A1, and EFNB1 in PB from three healthy children and B‐ALL cell lines (Nalm‐6, RS4;11 and SUP‐B15). (*p* < .05, *; *p* < .001, ***). EFNB1, NM_004429.5; BCL2A1, NM_001114735.2; CYBB, NM_000397.4

In order to verify the mRNA expression levels of *CYBB*, *BCL2A1*, and *EFNB1* in children B‐ALL, PB of three healthy children and B‐ALL cell lines (Nalm‐6, RS4;11 and SUP‐B15) were selected as experiment samples. The result of qRT‐PCR method showed that expression levels of *CYBB*, *BCL2A1*, and *EFNB1* were significantly lower in B‐ALL cell lines than the PB samples from three healthy children (Figure 8a–c). Perhaps they all played the role of tumor suppressor genes.

### 3.9 Immune cell infiltration

**FIGURE 9:**
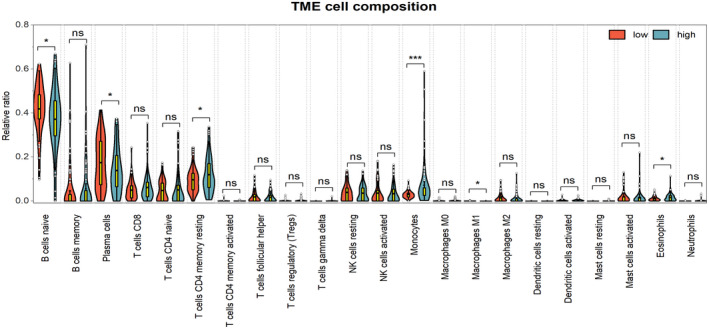
Relative percentage of 22 immune cell infiltration in high‐risk and low‐risk groups. T cells CD4 memory resting (*p* = .031), monocytes (*p* < .001), and eosinophils (*p* = .038) were all significantly abundant in high‐risk group

To understand the mechanism contributing to the poor prognosis, we compared the difference of 22 immune cells composition between high‐risk and low‐risk groups via CIBERSORT algorithm. Compared with low‐risk group, T cells CD4 memory resting (*p* = .031), monocytes (*p* < .001), and eosinophils (*p* = .038) occupied higher proportions of infiltration in high‐risk group. However, naïve B cells (*p* = .024), plasma cells (*p* = 0.043), and macrophages M1 (*p* = .031) were significantly more abundant in the low‐risk group than in the high‐risk group (Figure 9).

## 4 DISCUSSION

In this study, 112 co‐DEGs between normal B cells and children B‐ALL cells from two GEO datasets were identified by R software “limma” package, GO terms analysis mainly enriched in positive regulation of transcription by RNA polymerase II, protein binding, signal transduction, and zinc ion binding; KEGG analysis mainly enriched in pathways as follows: HIF‐1, PD‐1 checkpoint, TNF, Phospholipase D, Hippo, and MAPK, numerous researches have reported these signaling pathways were activated in the development progress of B‐ALL (Inaba & Mullighan, 2020; James et al., 2019; Tran & Hunger, 2020; Wellmann et al., 2004; Wunderlich et al., 2021; Yang et al., 2020). Besides, 10 key genes were screened by cytoHubba plugin in Cytoscape, among them, four key genes (*CYBB*, *BCL2A1*, *IFI30*, and *EFNB1*) were revealed to be associated with 5‐year OS of patients by KM and univariate Cox regression analysis. Then, *CYBB*, *BCL2A1*, and *EFNB1* were used to construct risk model via stepwise regression AIC and multivariate Cox regression analysis, the regression coefficient was −0.14097, 0.193929, and −0.22053, respectively. This result indicated the *CYBB* or *EFNB1* mRNA expression level was negatively associated with the 5‐year OS of patients, while *BCL2A1* mRNA expression level showed contrary correlation. Garcia‐Manero et al. showed that higher level of *CYBB* was associated with longer survival in AML or myelodysplastic syndromes (MDS) patients (Garcia‐Manero et al., 2012). EphB1, the receptor of EFNB1, functioned as a tumor suppressor in AML, EphB1 repression was associated with poor prognosis of pediatric AML (Kampen et al., 2015). Based on the median risk score, the patients were divided into low‐risk and high‐risk groups and 5‐year OS was calculated. Without exception, high‐risk patients had lower OS compared to low‐risk patients. Furthermore, ROC curve analysis was utilized to estimate the efficiency of prognostic risk model, the AUC was 0.729, and the result indicated that this three‐gene‐based prognosis signature had a strong ability to predict the 5‐year OS of CYAYs with B‐ALL.

To furtherly estimate the predictive value of three‐gene‐based signature, we investigated the correlation between the risk score and clinical features (age, gender, WBC count, CNS grade, MRD, *BCR‐ABL1*, and DNA Index), the result showed that no statistically significant relation existed between them. Additionally, to assess the independence of the signature, we compared the risk score with clinical features, the result of Cox regression indicated that risk score was confirmed as an independent factor for predicting the 5‐year OS of CYAYs with B‐ALL, and high risk score was negatively associated with the 5‐year OS.

To validate the mRNA levels, we used qRT‐PCR method to detect the mRNA levels of *CYBB*, *BCL2A1*, and *EFNB1* in samples from healthy donors and B‐ALL cell lines. The result showed that *CYBB*, *BCL2A1*, and *EFNB1* expression levels were all significantly lower in B‐ALL cells than controls. Similarly, Li et al. and Ramli et al. indicated that *CYBB* and *BCL2A1* expression levels were both lower in leukemia than normal controls (Li et al., 2009; Ramli et al., 2021).

Previous researches have shown CYBB acted as a driver to promote leukemia cell proliferation and suppress apoptosis (Abdul‐Aziz et al., 2019; Marlein et al., 2017), and regulated self‐renewal of leukemic stem cells. Irwin et al. suggested that CYBB mediated tyrosine kinase inhibitors (TKI) resistance (Irwin et al., 2015). *BCL2A1*, gene encoding a member of the BCL‐2 family protein, it could mediate drug resistance such as asparaginase resistance in B‐ALL (Chien et al., 2015), cytarabine and doxorubicin resistance in AML (Simpson et al., 2006), fludarabine and ABT‐737 resistance in chronic lymphocytic leukemia (CLL) cells (Morales et al., 2005; Olsson et al., 2007; Ottina et al., 2012; Vogler et al., 2009). In addition, BCL2A1 was a critical mediator of B‐cell survive and regulated by Spleen tyrosine kinase and Bruton tyrosine kinase, and was associated with advancement of hematological malignancies as well as solid tumor (Sochalska et al., 2016; Vogler, 2012). EFNB1 was a critical factor in stromal‐mediated support of hematopoiesis, and help to maintain the hematopoietic stem niche (Arthur et al., 2019). However, EphB1 as a tumor suppressor which mediated growth inhibition, apoptosis, and cycle arrest of EphB1‐expressing AML cells (Kampen et al., 2015). Eph/ephrin has been implied in tumorigenesis, metastasis, invasion, and drug resistance in some human cancers (Iwasaki et al., 2018; Kataoka et al., 2002; Vermeer et al., 2013).

The tumor microenvironment (TME) was an important factor in the development and progression of ALL, and immune cell infiltration was a component of TME (Cencini et al., 2021; Pastorczak et al., 2021; Simioni et al., 2021; Witkowski et al., 2020). For a better understanding the reasons contributing to poor prognosis, we evaluated the levels of 22 tumor‐infiltrating immune cells via CIBERSORT analysis. We found that six immune infiltrating cells, including naïve B cells, plasma cells and macrophages M1, T cells CD4 memory resting, and monocytes and eosinophils, were significantly associated with the prognostic model risk score, this result demonstrated there were different immune status between high‐risk and low‐risk groups. Similar results have been observed in several studies (Cencini et al., 2021; Pastorczak et al., 2021; Witkowski et al., 2020).

In the present study, it was found that the novel three‐gene signature‐related prognostic model had not been reported. More notably, as regards immune infiltrating cells in TME, we found significant difference existed between high‐risk and low‐risk patients. These findings could help better risk stratification of CAYAs with B‐ALL and guide individual treatment opinion. Additionally, the mRNA expression levels of three genes in B‐ALL cells were confirmed statistically different compared with controls by qRT‐PCR assay rather than relying on the database completely, this increased credibility of result.

However, there were several limitations in this study. First, the expression and the prognostic roles of CYBB, BCL2A1, and EFNB1 at protein level were not validated, further investigation was warranted in our clinical samples. Second, the underlying molecular mechanisms of CYBB, BCL2A1, and EFNB1 were not performed in vivo and in vitro experiments, further exploration may be valuable. Third, biological information of other DEGs might be overlooked. Fourth, because of limited sample size, the predictive efficiency of three‐gene‐based prognosis signature was not validated in external samples. These deficiencies will be the focus of our next studies.

## 5 CONCLUSION

In summary, on the basis of profiles (GSE48558 and GSE79533) and TARGET data matrix, a three‐gene risk score signature was constructed and the signature was an independent prognostic factor for CAYAs with B‐ALL; more importantly, immune cells infiltration containing T cells CD4 memory resting, monocytes, and eosinophils were highly abundant in high‐risk patients. To our knowledge, no such study has been proposed previously. Only detected mRNA expression, the patients are stratified into high risk and low risk, which significantly reduces the cost of sequencing and is more routine in practice. The information gained from this study help to assess the prognosis of CAYAs with B‐ALL and advance individualized treatment which will further improve the quality of life and the cure rate for CAYAs with B‐ALL.

## AUTHOR CONTRIBUTIONS

Liang Yu conceived and designed the study. Chunli Xiang and Jie Wu contributed equally to this work. Chunli Xiang and Jie Wu collected materials, performed data analysis, and drafted the manuscript. All authors read and approved the manuscript.

## CONFLICT OF INTEREST

The authors declare that they have no competing interest.

## ETHICAL APPROVAL

The study protocol was approved by the Huai'an First People's Hospital and was in accordance with the Declaration of Helsinki. IRB approval number: YX‐2021‐088‐01.

## Supporting information


Figure S1

## Data Availability

The data that support the findings of this study are available from the corresponding author upon reasonable request.
