# Supplementary figures and images for: Construction of three‐gene‐based prognostic signature and analysis of immune cells infiltration in children and young adults with B‐acute lymphoblastic leukemia
Source: Mol Genet Genomic Med. 2022 May 23;10(7):e1964. doi: 10.1002/mgg3.1964 (PMC9266608; doi:10.1002/mgg3.1964)

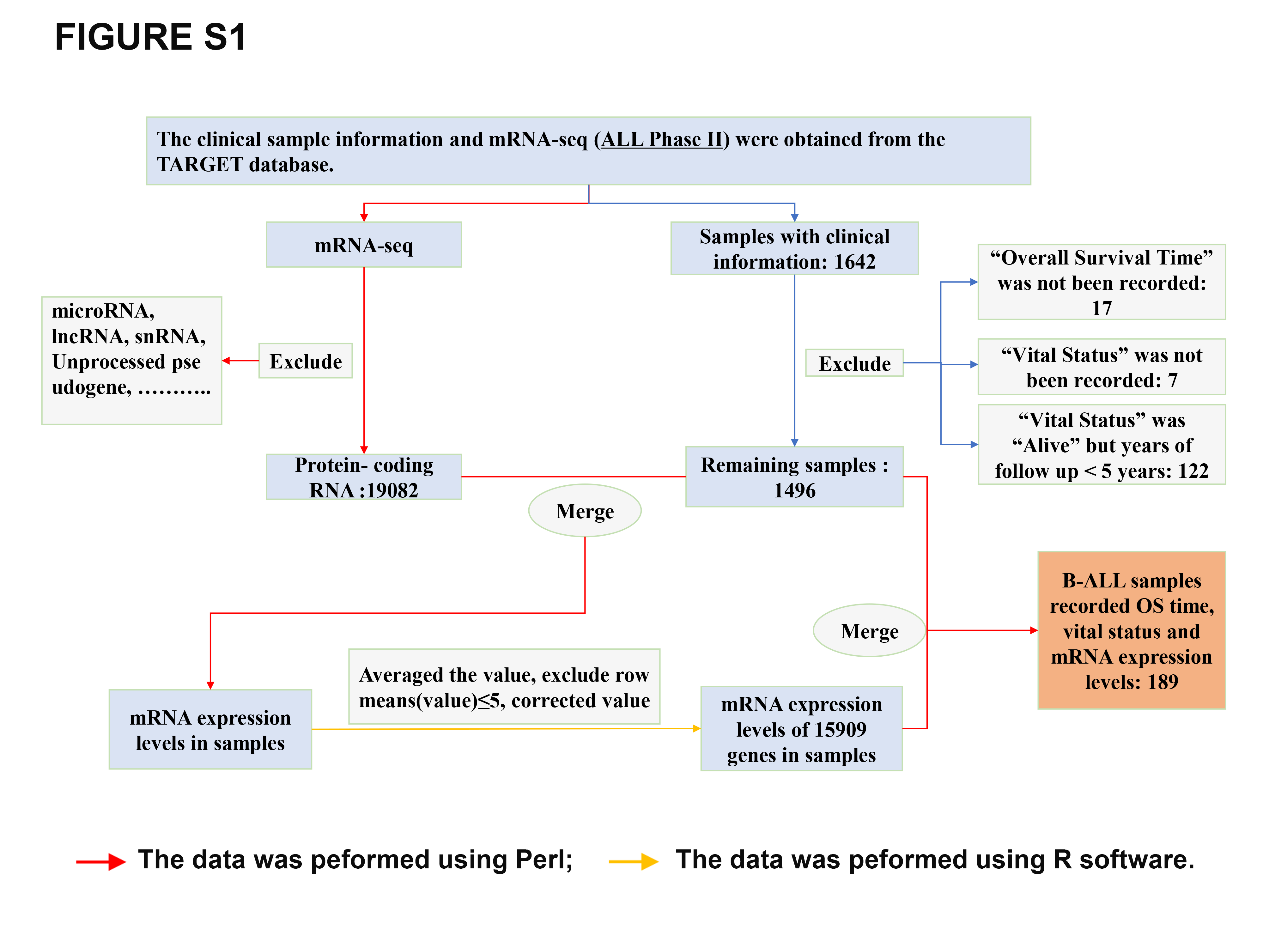


**FIGURE S1.** The specific screening process of 189 CAYAs with B-ALL samples.

Supplement: Supplementary file 1 — Figure S1 [file MGG3-10-e1964-s001.docx]
